# Efficacy and safety of subcutaneous venom immunotherapy in children: A 24‐year experience in a pediatric tertiary care center

**DOI:** 10.1111/pai.70195

**Published:** 2025-09-01

**Authors:** Mattia Giovannini, Francesco Catamerò, Marzio Masini, Federico Gelain, Elio Novembre, Simona Barni, Giulia Liccioli, Lucrezia Sarti, Leonardo Tomei, Benedetta Pessina, Claudia Valleriani, Chiara Marzi, Michela Baccini, Mohamed Shamji, Francesca Mori

**Affiliations:** ^1^ Department of Health Sciences University of Florence Florence Italy; ^2^ Allergy Unit, Meyer Children's Hospital IRCCS Florence Italy; ^3^ Immunology Laboratory, Meyer Children's Hospital IRCCS Florence Italy; ^4^ Department of Statistics, Computer Science and Applications “G. Parenti,” University of Florence Florence Italy; ^5^ NIHR Imperial Biomedical Research Centre London UK; ^6^ National Heart and Lung Institute, Imperial College London UK

**Keywords:** adverse reaction, anaphylaxis, children, hymenoptera, venom immunotherapy

## Abstract

**Background:**

Hymenoptera venom allergy is a significant cause of morbidity and mortality, also in pediatric patients, highlighting the importance of effective management through venom immunotherapy (VIT). This study aimed to evaluate the safety profile of VIT, identify factors associated with adverse reactions (ARs), assess the accuracy of insect identification and its impact on VIT extract selection, and determine treatment efficacy by analyzing ARs following re‐sting.

**Methods:**

The medical charts of patients followed up at the Allergy Unit of Meyer Children's Hospital IRCCS, Florence, Italy, who completed a VIT cycle between 1997 and 2021 were retrospectively analyzed. VIT extract selection was guided by a diagnostic workup following the European Academy of Allergy and Clinical Immunology guidelines and the Italian Consensus on Hymenoptera venom allergy management. We implemented a cluster protocol and adjusted it as needed for ARs during VIT.

**Results:**

Fifty‐eight patients, from a total of 60 VIT (2 patients underwent VIT for both Vespula and Polistes) were included, using the following extracts: 17 *Apis mellifera* (28.4%), 20 *Vespula* (33.3%), 20 *Polistes* (33.3%), and 3 *Vespa crabro* (5.0%). Upon the 3739 injections administered, 355 ARs (9.5%) occurred: local reactions (LRs), 306 (8.2%); extended local reactions (ELRs), 34 (0.9%); and systemic reactions (SRs), 15 (0.4%). The build‐up phase was associated with a higher number of ARs and LRs compared with the maintenance phase during VIT (*p* < .0001), normalized by the number of injections. No other significant factors related to the risk of developing any ARs were highlighted. The highest SR rate was found in the VIT for *Polistes*, with no significant differences in AR proportions among the venom extracts. Thirty patients reported 51 re‐stings following VIT, with only 2 of 51 (3.9%) resulting in SRs. These reactions occurred in individuals stung by a different Hymenoptera species from the one targeted during the VIT.

**Conclusion:**

Cluster protocol VIT is safe and effective in pediatric patients.

**Key message**

The build‐up phase was associated with a higher frequency of allergic reactions than the maintenance phase (normalized by the number of injections). Despite initial systemic reactions, venom immunotherapy (VIT) proved effective upon re‐sting. VIT for *Polistes* had the highest rate of SRs, even if VIT proved safe.

## 1 INTRODUCTION

Hymenoptera venom allergy is a significant cause of morbidity and mortality in both adults and children.^1^ Hymenoptera venom contains allergenic components, with phospholipases A1 and A2 being the primary sensitizing allergens responsible for anaphylaxis.^2^, ^3^, ^4^ The venom sensitization rate of Hymenoptera in children is about 4%,^5^ and the risk of systemic reactions (SRs) is estimated at approximately 1%.^6^ Anaphylactic reactions to Hymenoptera venom represent 20% of the total anaphylaxis cases in the pediatric age, and fatal events have been reported from 5 years of age.^7^, ^8^ Fatal anaphylaxis due to Hymenoptera stings is possible even on first exposure, but definitive risk factors for SRs in children are unclear.^9^

Currently, venom immunotherapy (VIT) is the only effective treatment for venom allergy, and it is recommended for individuals who have experienced SRs to stings and tested positive for venom‐specific allergens.^10^, ^11^, ^12^, ^13^, ^14^, ^15^ Whilst most allergic reactions during VIT are localized, SRs occur at a relatively low frequency of 0.1%–0.2%.^16^, ^17^

Although the safety profile of VIT has been extensively studied in adults,^18^, ^19^, ^20^ data on adverse reactions (ARs) in children remain limited.^21^ Furthermore, predictive risk factors for ARs during VIT have not been fully elucidated.

The present study aimed to evaluate both the frequency and severity of ARs during VIT in a cohort of children and to identify potential predictors of these reactions. Furthermore, we report the first pediatric cohort in which data on VIT for *Polistes* species were analyzed, addressing the scarcity of information regarding VIT for this Hymenoptera. Finally, we describe the outcomes of re‐stings in children treated with VIT.

## 2 METHODS

### 2.1 Study population and diagnosis of venom allergy

The medical charts of patients followed up at the Allergy Unit of Meyer Children's Hospital IRCCS, Florence, Italy, in the period between 1997 and 2021, who underwent a complete 5‐year cycle subcutaneous VIT were retrospectively analyzed. These charts were unique for each patient and consisted of VIT schedules, ARs during VIT, and treatments administered after ARs.

The diagnosis of Hymenoptera venom allergy and the choice of VIT extract were based on a diagnostic workup conducted according to the European Academy of Allergy and Clinical Immunology guidelines^22^ and the Italian Consensus on the Management of Allergy to Hymenoptera venom.^23^ We routinely performed skin prick tests (SPTs) followed by intradermal tests (IDTs). For SPT concentrations, 100 μg/mL were utilized, except for individuals with severe reactions, who were tested with lower concentrations (0.1–10 μg/mL) at first. A wheal ≥3 mm in diameter after 15 min was deemed positive, with histamine as the positive control and saline solution as the negative control. For IDTs, concentrations ranging from 0.001 to 1 μg/mL were used by intradermally injecting a 0.02‐mL volume of allergen extract in order to produce a wheal of approximately 3 mm in diameter. The reading was considered positive when the wheal increased by ≥3 mm after 15–20 min or a doubling of the initial wheal diameter was obtained. IDT was performed in all patients, even when the SPT yielded a positive result, to identify the cutaneous endpoint for VIT.^24^ Moreover, serum‐specific immunoglobulin E (sIgE) against the most common venom allergens was measured by the ImmunoCAP (Thermo Fisher Scientific, Uppsala, Sweden) technique. We collected patients' personal and family histories of atopy, allergic rhinoconjunctivitis (RC), asthma, atopic dermatitis, and venom allergy. The diagnoses of RC and asthma were established following international guidelines.^25^, ^26^

### 2.2 VIT

VIT was performed using a cluster protocol^27^ adapted according to VIT progress for any AR, comprising a build‐up phase followed by a maintenance phase. During the build‐up phase, two administrations with increasing volumes were typically performed in 1 day, starting with a venom concentration determined by the endpoint. The concentrations were increased weekly until a target dose of 100‐μg dose was reached consistently across all Hymenoptera species.^28^ In the maintenance phase, a single 100‐μg dose was administered simultaneously.^21^ This procedure was repeated every 4 weeks during the first year and gradually extended to intervals of 6–8 weeks in subsequent years, for a total of 5 years of treatment.

### 2.3 ARs

ARs were categorized as follows: (1) local reactions (LRs): characterized by pain, erythema, and edema confined to the sting site, typically resolving within 24 h; (2) extended local reactions (ELRs): characterized by edema extending over 10 cm from the sting site lasting more than 24 h; (3) SRs: predominantly IgE‐mediated,^2^ characterized by systemic manifestations appearing within minutes post‐sting, potentially involving multiple organ systems.

The severity of SR pre‐VIT or upon re‐sting was assessed using Muller's classification,^3^ and Ring's classification^29^ was employed to assess the severity of SRs during VIT (Table 1).

**TABLE 1:** Muller's and Ring's classification divided by the grade (severity) of the reaction.

Muller's classification	
Grade I	Generalized urticaria, pruritus, malaise, anxiety
Grade II	Any grade I plus two or more additional symptoms (angioedema, chest tightness, nausea, vomiting, diarrhea, abdominal pain, dizziness)
Grade III	Any grade II plus two or more additional symptoms (dyspnea, wheezing, laryngeal stridor, dysarthria, hoarseness, asthenia, confusion, impending sense of doom)
Grade IV	Any grade III plus two or more additional symptoms (decreased blood pressure, collapse, loss of consciousness, incontinence, cyanosis)
Ring's classification	
Grade I	Generalized cutaneous symptoms (erythema, urticaria, angioedema)
Grade II	Mild to moderate pulmonary, cardiovascular, and/or gastrointestinal symptoms
Grade III	Anaphylactic shock, loss of consciousness
Grade IV	Cardiac arrest, apnea

Following VIT completion, patients were contacted by phone to ascertain if they were restung and their subsequent reaction.

### 2.4 Statistical analysis

We summarized continuous variables using the median and interquartile range (IQR) and expressed categorical variables as absolute frequencies and corresponding percentages.

We aimed to identify potential factors associated with the risk of developing ARs (LRs, ELRs, and SRs) during VIT. The analysis considered demographic and clinical variables, such as sex, age at the start of VIT, family history of atopy, personal history of atopy, and grade of the primary reaction before VIT. Since each patient may have received a different number of injections during the build‐up and maintenance phases, we reported ARs data per patient. Patients who experienced at least one AR (LRs, ELRs, or SRs) were assigned a value of 1. We then modeled this binary outcome using multiple logistic regression to assess the relationship with the abovementioned factors. Only complete cases were included in regression models. Observations with missing values in any of the variables used were excluded from the analysis.

We also assessed the differences in the number of ARs (LRs, ELRs, and SRs) occurring during the build‐up and maintenance phases. To account for the varying number of injections administered to the same patient in the two phases, we normalized the number of ARs (LRs, ELRs, and SRs) by the number of injections in each phase. The results were then compared using the Wilcoxon signed‐rank test for non‐normally distributed paired data.

We compared the number of ARs (LRs, ELRs, and SRs) (normalized by the total number of injections) across the different types of extracts used for VIT (*Apis mellifera*, *Polistes*, *Vespa crabro*, *Vespula*) using the Kruskal–Wallis test for non‐normally distributed data, followed by the Mann–Whitney test with Bonferroni correction.

We qualitatively described the re‐stinging experiences following the initiation of VIT and assessed their concordance with the extract type used during treatment.

## 3 RESULTS

### 3.1 Baseline characteristics and identification of the stinging insects

Data from 58 patients (51 male, 87.9%) under 18 years of age (median age 9.4 years, IQR = 7 years) were retrospectively retrieved. A total of 60 VITs were conducted in 58 patients (2 of whom underwent dual VIT for both *Vespula* and *Polistes*), distributed as follows: 20 for *Vespula* (33.3%), 20 for *Polistes* (33.3%), 17 for *Apis mellifera* (28.4%), and 3 for *Vespa crabro* (5.0%). The median VIT duration was 5.4 years (IQR = 1.8 years), and the median number of injections per patient was 63.4 (IQR = 23.6). Twenty‐one patients (21/44, 47.7%; data were not available for 14 patients) had a positive family history of atopy, and 16 patients (16/58, 27.6%) presented atopic manifestations as follows: rhinoconjunctivitis (11/58, 18.9%), asthma (4/58, 6.8%), food allergy (4/58, 6.8%), and atopic dermatitis (3/58, 5.2%). Among patients who underwent SPTs (47/58) and IDTs (47/58) before starting VIT, SPTs were positive in 26/47 patients (55.3%) and IDTs were positive in 31/38 (81.6%) patients. The median value of sIgE against Hymenoptera venom was 12.0 kU/L (IQR = 32.7 kU/L) before starting VIT, while, at the end of VIT, the median value was 1.92 kU/L (IQR = 4.9 kU/L).

Patients started VIT after experiencing the following ARs (for one patient, it was not possible to properly assess the reaction): ELRs (1/57, 1.7%); SRs (56/57, 98.3%). According to the Muller severity scale, patients' reactions were clustered as follows: M1 3/57 (5.3%); M2 17/57 (29.8%); M3 33/57 (57.9%); M4 3/57 (5.3%). The demographic and clinical characteristics are summarized in Table 2. In 22/47 (46.8%) patients, Hymenoptera were identified (data from clinical records were unavailable for 11 patients), in 1/47 (2.1%) remained unrecognized, while in 24/47 (51.1%) were identified as members of the *Vespidae* family but were not further characterized. Among the patients treated with VIT, concordance between the identified insect species and the extract used was observed in the following patients: 14/15 patients (93.3%) treated with *Apis mellifera* extract correctly identified the species, 5/16 patients (31.2%) treated with *Vespula* extract, 3/3 patients (100%) treated with *Vespa crabro* extract, and none of the 10 patients (0%) treated with *Polistes* extract. Additionally, 24/32 (75.0%) patients receiving *Vespidae* extract were able to identify the insect as belonging to this family but without distinguishing between *Vespula* and *Polistes*.

**TABLE 2:** Patient characteristics at the start of venom immunotherapy.

	*n* = 58
Male sex – *n* (%)	51 (87.9)
Age at the beginning of VIT, *years* – median (IQR)	9.4 (7)
Family history of atopy – *n* (%)	21 (47.7)
Atopy – *n* (%)	
Rhinoconjunctivitis	11 (18.9)
Asthma	4 (6.8)
Food allergy	4 (6.8)
Atopic dermatitis	3 (5.2)
Muller severity scale patients' reactions – *n* (%)	
M1	3 (5.3)
M2	17 (29.8)
M3	33 (57.9)
M4	3 (5.3)

Abbreviations: IQR, interquartile range; VIT, venom immunotherapy.

### 3.2 ARs during VIT and potential associated factors

During the study period, a total of 3739 injections were administered. ARs were documented in 355 (9.5%) patients. Specifically, 306 (8.2%) were LRs, 34 (0.9%) ELRs, and 15 (0.4%) SRs. We evaluated sex, age at the beginning of VIT, family history of atopy, personal history of atopy, and severity of the reaction before VIT as potential factors associated with ARs (LRs, ELRs, and SRs) during VIT.

#### 3.2.1 Sex

In our cohort, 3230 injections (86.4%) were administered to males, with 300 ARs reported (9.3%). Specifically, LRs occurred in 258 cases (86%), ELRs in 33 cases (11%), and SRs in 9 cases (3%). Sex was not associated with the development of ARs, LRs, or ELRs during VIT (Table 3).

**TABLE 3:** Multiple logistic regression models for risk factors associated with ARs, LRs, ELRs, and SRs during VIT.

	OR	Lower 95% CI	Upper 95% CI	*p*‐Value
ARs				
Intercept	21.768	0.666	711.626	.083
Sex	0.759	0.069	8.302	.821
Age at the beginning of VIT	0.868	0.671	1.123	.282
Atopy	0.856	0.124	5.902	.874
Severity of the reaction pre‐VIT	1.059	0.178	6.307	.950
Family history of atopy	1.594	0.252	10.088	.620
LRs				
Intercept	12.929	0.457	366.123	.134
Sex	0.692	0.064	7.472	.762
Age at the beginning of VIT	0.884	0.695	1.124	.313
Atopy	0.996	0.152	6.513	.997
Severity of the reaction pre‐VIT	1.417	0.266	7.561	.683
Family history of atopy	1.816	0.316	10.445	.504
ELRs				
Intercept	0.406	0.014	12.121	.603
Sex	1.535	0.132	17.807	.732
Age at the beginning of VIT	0.838	0.657	1.07	.157
Atopy	5.415	0.824	35.609	.079
Severity of the reaction pre‐VIT	3.069	0.455	20.701	.25
Family history of atopy	0.301	0.046	1.98	.212
SRs				
Intercept	0.881	0.007	104.389	.958
Sex	0.051	0.002	1.224	.066
Age at the beginning of VIT	0.933	0.643	1.353	.714
Atopy	7.774	0.538	112.394	.132
Severity of the reaction pre‐VIT	0.17	0.011	2.727	.211
Family history of atopy	3.291	0.228	47.484	.382

*Note*: Estimates are expressed as odds ratios with 95% confidence intervals (CI) and corresponding *p*‐values. Models include the following covariates: Sex (female reference), age at initiation of VIT (continuous, in years), atopy (“none” as reference), severity of pre‐VIT reaction (SR grading: “low” [M1–M2] as reference), and family history of atopic disease (“absent” as reference). Patients with missing data were excluded using complete‐case analysis. Significance level was set at .05.

Abbreviations: AR, adverse reaction; CI, confidence interval; ELR, extended local reaction; LR, local reaction; SR, systemic reaction; VIT, venom immunotherapy.

#### 3.2.2 Age at the beginning of VIT

In patients under 6 years of age, we administered 772 (20.6%) injections, with 104 ARs: LRs were 93 (93/104; 89.4%), ELRs were 10 (10/104; 9.6%), and SRs were reported in only 1 patient (1/104; 0.9%). In patients aged between 6 and 11 years of age, we administered 1947 (52.1%) injections, with 160 ARs: LRs were 134 (134/160; 83.8%), ELRs were 19 (19/160; 11.9%), and SRs were reported in 7 patients (7/160; 4.3%). In patients older than 12 years of age, we administered 1020 (27.2%) injections, with 91 ARs: LRs were 79 (79/91; 86.8%), ELRs were 5 (5/91; 5.5%), and SRs were reported in 7 patients (7/91; 7.7%). Age was not statistically significantly associated with the possibility of developing any ARs or LRs, ELRs, or SRs (Table 3).

#### 3.2.3 Family history of atopy

There was an established family history of atopy in 21 patients (21/44, 47.7%; data for 14 patients were not found in the electronic chart) In those patients, 1404 injections were administered, with 139 ARs reported. LRs represented the majority of cases (129/139, 92.8%). ELRs were registered in 3/139 cases (2.2%) and SRs in 7/139 cases (5.0%). A family history of atopy was not identified as a factor associated with the development of ARs, LRs, ELRs, or SRs (Table 3).

#### 3.2.4 Atopy

Sixteen patients (16/58, 27.6%) with atopy were identified, and this group received a total of 1055 injections, with 98 ARs reported. These ARs were distributed as follows: 84 LRs (85.7%), 10 ELRs (10.2%), and 4 SRs (4.1%). Atopy was not associated with the development of any ARs, LRs, or ELRs (Table 3).

#### 3.2.5 Severity of the reaction pre‐VIT

In 3 patients who experienced an M1 reaction before starting VIT, 228 injections were administered, with 37 ARs reported: 35 LRs (15.3%), 2 ELRs (0.9%), and no SRs. In 17 patients with an M2 reaction before starting VIT, 1063 injections were administered, resulting in 106 ARs: 90 LRs (84.9%), 7 ELRs (6.6%), and 9 SRs (8.5%). In 33 patients who reported an M3 reaction before starting VIT, 2201 injections were administered, with 186 ARs observed: 159 LRs (85.5%), 22 ELRs (11.8%), and 5 SRs (2.7%). At last, in 3 patients with an M4 reaction before starting VIT, 191 injections were administered, with 26 ARs reported: 22 LRs (84.6%), 3 ELRs (11.5%), and 1 SR (3.9%). The severity of the reaction before VIT, categorized as “low,” i.e., M1 and M2, and “high,” i.e., M3 and M4, was not identified as a factor associated with the development of any ARs (Table 3).

### 3.3 Differences between the build‐up and maintenance phases

**FIGURE 1:**
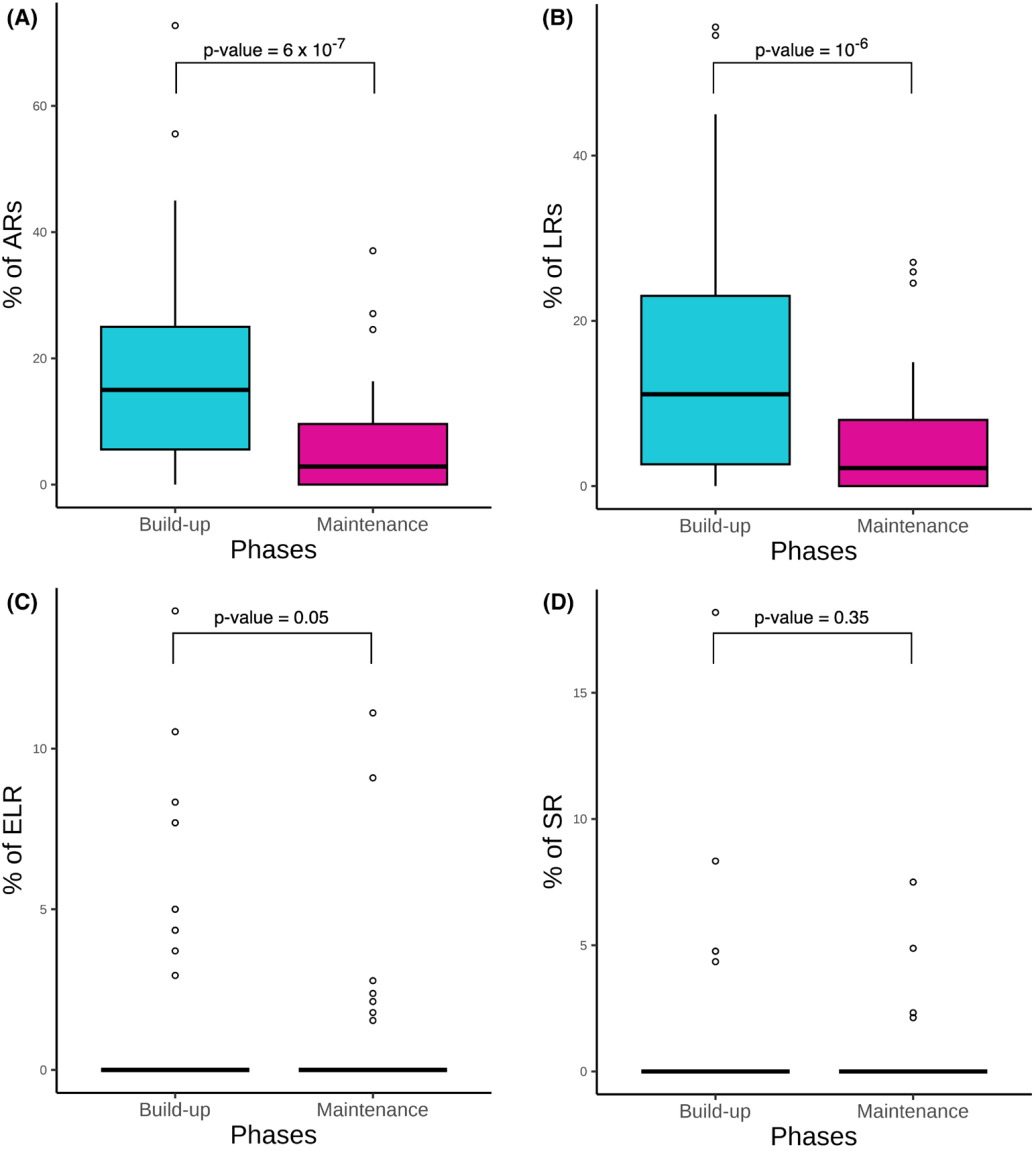
Differences between the build‐up and maintenance phase for adverse reactions (ARs) (A), local reactions (LRs) (B), extended local reactions (ELRs) (C), and systemic reactions (SRs) (D). To account for the varying number of injections administered to the same patient in the two phases, we normalized the number of ARs (LRs, ELRs and SRs) by the number of injections in each phase. The results were then compared using the Wilcoxon signed‐rank test for non‐normally distributed paired data. The number of ARs (*p* < .0001), in particular LRs (*p* < .0001), was significantly higher during the build‐up phase compared with the maintenance phase. No significant differences were observed for ELRs (*p* = .5) and SRs (*p* = .35).

During the build‐up phase, 1120 injections were administered, with 194 ARs reported. LRs represented the majority of cases, with 170/194 (87.6%) reactions; ELRs were reported in 16/194 (8.2%) cases and SRs only in 8/194 (4.1%) patients. On the other hand, during the maintenance phase, 2619 injections were administered, with 161 ARs reported, divided as follows: LRs, 136/161 (84.5%); ELRs 18/161 (0.7%); SRs 7/161 (0.3%). The number of ARs (*p* < .0001), in particular LRs (*p* < .0001), normalized by the number of injections, was significantly higher during the build‐up phase compared with the maintenance phase. In contrast, no significant differences between the two phases were observed for ELRs (*p* = .5) or SRs (*p* = .35) (Figure 1).

### 3.4 Type of extract used for VIT

The reactions were investigated depending on the different types of extracts used for VIT. *Apis mellifera* extract was administered in 1030 injections, with 82 ARs (8%) divided as follows: LRs 66/82 (80.5%); ELRs 12/82 (14.6%); and SRs 4/82 (4.9%). *Vespula* extract was administered in 1329 injections, with 153 ARs (11.5%), divided as follows: LRs 138/153 (90.2%); ELRs 10/153 (6.5%); and SRs 5/153 (3.3%). *Polistes* extract was administered in 1175 injections, with 113 ARs (9.6%) divided as follows: LRs 95/113 (84.1%); ELRs 12/113 (10.6%); and SRs 6/113 (5.3%). *Vespa crabro* extract was administered in 205 injections, with just 7 LRs (3.4%) reported. (Table 4).

**TABLE 4:** Number of total adverse reactions, local reactions, extended local reactions, and systemic reactions grouped by different types of extract used for venom immunotherapy.

	Total adverse reactions	Local reactions	Extended local reactions	Systemic reactions
*Apis mellifera*	82	66 (80.5%)	12 (14.6%)	4 (4.9%)
(*n* = 1030)	[8.0%]^a^	[6.4%]^a^	[1.2%]^a^	[0.4%]^a^
*Vespula*	153	138 (90.2%)	10 (6.5%)	5 (3.3%)
(*n* = 1329)	[11.5%]^a^	[10.4%]^a^	[0.8%]^a^	[0.4%]^a^
*Polistes*	113	95 (84.1%)	12 (10.6%)	6 (5.3%)
(*n* = 1175)	[9.6%]^a^	[8.1%]^a^	[1.0%]^a^	[0.5%]^a^
*Vespa crabro*	7	7 (100.0%)	0 (0.0%)	0 (0.0%)
(*n* = 205)	[3.4%]^a^	[3.4%]^a^	[0.0%]^a^	[0.0%]^a^

*Note*: *n*, indicates the total number of injections administered for that type of extract.

^a^ The number of reactions out of the total injections administered with that specific extract during venom immunotherapy.

No statistically significant differences were reported according to the extract used for VIT for the rate of ARs (*p* = .20) or a specific type of reaction (LRs: *p* = .22; ELRs: *p* = .66; SRs: *p* = .82).

### 3.5 Re‐stings

Thirty patients experienced 51 re‐stings, with 15 stung twice and three stung thrice. The median time to re‐sting was 4.3 years (IQR = 3.3 years) from the start of VIT. LRs were reported in 41 patients (41/51; 80.4%), followed by ELRs in 8 patients (8/51; 15.7%), and SRs in two cases (2/51; 3.9%). Concordant re‐stings (patients stung by the same species/family of Hymenoptera for which they underwent VIT) were reported in 30 patients: 4/30 (13.3%) were due to *Apis mellifera* (4 LRs, 0 ELR, and 0 SR), 26/30 (86.7%) were due to Hymenoptera from the *Vespidae* family (22 LRs, 4 ELRs, and 0 SR). Discordant re‐stings (patients stung by a species/family of Hymenoptera different from the one for which they underwent VIT) were reported in 13 patients: 5/13 (38.5%) were due to *Apis mellifera* (5 LRs, 0 ELR, and 0 SR), 8/13 (61.5%) were due to Hymenoptera from the *Vespidae* family (5 LRs, 1 ELRs, and 2 SRs of grade M2). SRs occurred in a patient who had undergone VIT for *Polistes* and was stung by *Vespa crabro* 3 years after starting VIT and in another patient who had undergone VIT for *Apis mellifera* and was stung by a vespid 4 years after beginning VIT. Lastly, the unidentified re‐stings were 8 (5 LRs and 3 ELRs) (Table 5).

**TABLE 5:** Number of local reactions, extended local reactions, and systemic reactions occurring after a concordant or discordant re‐sting following the completion of venom immunotherapy, categorized by the identified Hymenoptera species.

	Concordant re‐stings			Discordant re‐stings		
	(*n* = 30)			(*n* = 13)		
	LRs (*n* = 26)	ELRs (*n* = 4)	SRs (*n* = 0)	LRs (*n* = 10)	ELRs (*n* = 1)	SRs (*n* = 2)
*Apis mellifera*	4 (13.3%)	0 (0.0%)	0 (0.0%)	5 (38.5%)	0 (0.0%)	0 (0.0%)
*Vespidae family*	22 (73.3%)	4 (13.3%)	0 (0.0%)	5 (38.5%)	1 (7.7%)	2 (15.4%)

Abbreviations: ELR, extended local reaction; LR, local reaction; SR, systemic reaction.

## 4 DISCUSSION

In this 24‐year follow‐up study, we aimed to evaluate the safety and efficacy of VIT in a pediatric cohort by analyzing the incidence of ARs during VIT using a cluster protocol and identifying potential factors associated with the development of ARs (LRs, ELRs, and SRs) during VIT. The results showed that the cluster protocol represents a safe and effective treatment in children, with a low rate of SR (0.4% in relation to the total number of doses administered) and none requiring epinephrine. Moreover, LRs were identified in around 10% of injections, which seems higher than what was reported in other studies adopting a traditional VIT protocol.^30^, ^31^ However, the percentages of SRs and ELRs were similar.^30^, ^31^ This difference in the incidence of LRs might be attributed to the faster up‐dosing of the cluster protocol compared with the traditional protocol, as the former could predispose patients to a higher rate of LRs without increasing the risk of ELRs or SRs. These findings align with the literature on rush protocols, which shows a significantly higher percentage of SRs.^32^, ^33^ Our study suggests that, despite the increased occurrence of mild LRs, the cluster protocol appears to be an optimal approach for administering VIT, as it balances faster treatment outcomes with a high safety profile. Although the cluster protocol was time‐consuming for the patient, it did not require venous access placement, making it significantly better tolerated by patients and less resource‐intensive in terms of healthcare assistance than rush/ultra‐rush protocols. Moreover, compared with the traditional protocol, the clustered protocol offers advantages in terms of a shorter time to reach the maintenance dose and a reduced number of medical visits, a particularly relevant benefit for patients living in rural areas or outside urban centers who need to travel to access VIT.

Furthermore, the build‐up phase was associated with a statistically significant increase in ARs, specifically LRs, consistent with findings previously reported in the literature.^9^, ^30^

Being male appears to be associated with a lower risk of developing ARs, compared with females, consistent with what was already reported by Albuhairi et al., who showed a statistically significant difference in a more homogeneous sample than ours, which showed a male predominance.^31^ Interestingly, Kohli‐Wiesner et al. reported that female sex was a significant risk factor for the development of SR during VIT.^34^

Age at the beginning of VIT and family history of atopy did not show any statistically significant difference. These results are aligned with what is already reported in the literature.^30^

Approximately 30% of patients in our cohort had a concurrent history of atopy, but no statistically significant association was observed, in line with most findings in the literature. Conversely, in the study by Gür Çetinkaya et al.,^30^ asthma emerged as a significant predictor of SRs during VIT. The severity of SRs before VIT was not a significant risk factor in our analysis; however, this parameter has not been further explored in the literature.

Unlike most studies, which primarily focused on *Apis mellifera*, *Vespula*, and *Vespa crabro* extracts, our data revealed the highest rate of SRs, relative to total doses administered during VIT, for *Polistes* (0.5%) but no statistically significant differences in ARs proportions observed among the different venom extracts were highlighted.

Furthermore, the extremely low rate of SRs upon re‐stings (two instances of M2 SRs occurred in discordant re‐stings) demonstrated the effectiveness of VIT. After VIT, in concordant re‐stings, we described only LRs or ELRs, which had an absolute benefit on the patient's safety and quality of life.

Our data revealed that patients could easily identify *Apis mellifera* and *Vespa crabro* but struggled to differentiate *Vespula* from *Polistes* before and after VIT's start. Despite this, VIT demonstrated efficacy, deeply modifying the allergy's natural history.

Although our study provides valuable new insights, it has several limitations. The retrospective nature limited proper data collection, especially regarding patients' medical and family histories as well as the fact that different venom extracts were used during different study periods upon their availability in the hospital for treatment. Moreover, recall bias is a general limitation of retrospective studies based on clinical documentation. Patients may have confused or misidentified the stinging species during or after VIT, making it difficult to accurately assess the efficacy of VIT against a particular venom type. However, we presented images of Hymenoptera species to minimize misunderstandings, and sIgE and molecular diagnosis guided our choice for final identification. Finally, the sample was not equally distributed, with the male proportion higher than the female one, limiting the assumption concerning sex as a potential factor associated with ARs during VIT. Still, at the same time, Hymenoptera venom allergy is not very common in the pediatric age, thus limiting the possibility of designing new studies.

Despite these limitations, the study benefited from the longest clinical follow‐up ever analyzed in children and involved one of the largest cohorts of pediatric patients. Given the limited number of studies on ARs to VIT in children, this study is important for demonstrating the efficacy and safety of VIT in the pediatric population, a key treatment option that significantly improves children's quality of life.^35^ Moreover, it included data on VIT with *Polistes* venom, which are scarcely represented in the existing literature.

## 5 CONCLUSION

The present study confirmed that cluster protocol VIT is safe and effective in pediatric patients, with a low rate of SRs. The build‐up phase was associated with a higher frequency of ARs, while factors such as sex, age, atopy, and type of venom extract showed no significant impact. VIT with *Polistes* venom had the highest SR rate, requiring further validation. Despite initial SRs, VIT demonstrated indisputable efficacy upon re‐stinging, underscoring its value as an essential therapy for eligible patients.

## AUTHOR CONTRIBUTIONS

**Mattia Giovannini:** Conceptualization; writing – original draft; methodology; writing – review and editing; supervision. **Francesco Catamerò:** Writing – original draft; methodology; writing – review and editing. **Marzio Masini:** Investigation; visualization. **Federico Gelain:** Data curation. **Elio Novembre:** Supervision. **Simona Barni:** Writing – review and editing; supervision. **Giulia Liccioli:** Writing – review and editing; supervision. **Lucrezia Sarti:** Writing – review and editing; supervision. **Leonardo Tomei:** Writing – review and editing; supervision. **Benedetta Pessina:** Writing – review and editing; supervision. **Claudia Valleriani:** Data curation; software. **Chiara Marzi:** Methodology; formal analysis; data curation. **Michela Baccini:** Formal analysis; methodology; data curation. **Mohamed Shamji:** Supervision; writing – review and editing. **Francesca Mori:** Conceptualization; writing – review and editing; supervision; resources.

## FUNDING INFORMATION

The authors declare they have not received financial support.

## CONFLICT OF INTEREST STATEMENT

MG reports personal fees from Sanofi, Thermo Fisher Scientific.

## PEER REVIEW

The peer review history for this article is available at https://www.webofscience.com/api/gateway/wos/peer‐review/10.1111/pai.70195.

## ETHICS STATEMENT

Written informed consent was obtained from parents for all procedures performed. The code of the event report issued by Meyer Children's Hospital IRCCS is IR904‐23‐65255.
